# Serum levels of galactose-deficient IgA are elevated in patients with IgA nephropathy but do not correlate to disease activity or progression

**DOI:** 10.1186/s12882-023-03198-y

**Published:** 2023-06-07

**Authors:** Sigridur Elíasdóttir, Alina Khramova, Aso Saeed, Gregor Guron, Roberto Boi, Johan Mölne, Kerstin Ebefors, Jenny Nyström

**Affiliations:** 1 Department of Molecular and Clinical Medicine/Nephrology, Institute of Medicine, The Sahlgrenska Academy at the University of Gothenburg, Gothenburg, Sweden; 2 Department of Physiology, Institute of Neuroscience and Physiology, The Sahlgrenska Academy at the University of Gothenburg, Gothenburg, Sweden; 3 Institute of Biomedicine, Department of Laboratory Medicine, University of Gothenburg, Gothenburg, Sweden

**Keywords:** IgA nephropathy, Chronic kidney disease, Prognosis, Biomarkers, Albuminuria, Gd-IgA

## Abstract

**Introduction:**

IgA nephropathy (IgAN) is the most common glomerulonephritis globally. Because of the heterogeneity of the disease prognostic biomarkers are highly needed.

**Aim:**

To investigate associations between galactose-deficient IgA1 (Gd-IgA1) concentrations in plasma and urine and disease activity and progression in patients with IgAN.

**Methods:**

Serum and urine samples were collected at the time of kidney biopsy (baseline) in patients with IgAN (n = 40) and analysed for Gd-IgA1. Patients with chronic kidney disease (CKD) without IgAN (n = 21) and healthy controls (n = 19) were examined as controls. In 19 patients with IgAN, analyses of Gd-IgA1 were repeated after a median follow up time of approximately 10 years.

**Results:**

Serum Gd-IgA1 and Gd-IgA1:IgA were significantly elevated at the time of kidney biopsy in patients with IgAN compared to patients with non-IgAN CKD and healthy controls (p < 0.001). Urinary Gd-IgA1:creatinine was significantly elevated in patients with IgAN compared to patients with non-IgAN CKD. Neither serum Gd-IgA1, nor serum Gd-IgA1:IgA, correlated significantly to estimated GFR, urine albumin:creatinine (UACR), or blood pressure, at baseline. Serum Gd-IgA1 and Gd-IgA1:IgA at time of biopsy did not correlate significantly to annual changes in eGFR or UACR during follow up. In patients with IgAN, serum Gd-IgA1 decreased significantly over time during approximately 10 years of follow up (Δ-20 ± 85%, p = 0.027). Urinary Gd-IgA1:creatinine showed a strong positive correlation to UACR in patients with IgAN and likely reflected unspecific glomerular barrier injury.

**Conclusion:**

Although serum Gd-IgA1 and the Gd-IgA1:IgA ratio were significantly elevated in patients with IgAN at the time of kidney biopsy they were not related to disease activity or progression in this patient cohort.

## Introduction

IgA nephropathy (IgAN) is the most common glomerulonephritis globally and an important cause of end stage renal disease (ESRD) [1, 2]. The pathophysiology of IgAN is intricateand currently the ”four-hit hypothesis” is the most widely accepted theory [3]. According to this hypothesis, it is not sufficient to have increased plasma levels of galactose deficient IgA1 (Gd-IgA1) but autoantibodies must also bind to Gd-IgA1 molecules and form complexes that deposit in the mesangium and initiate an inflammatory cascade that ultimately causes glomerular injury [3]. A major conundrum in IgAN is that the clinical course is very heterogenous. This heterogeneity makes it difficult to study IgAN and to make treatment decisions in individual patients although risk stratification tools have been developed [4]. It is therefore important to gain further knowledge about the natural course of the disease and to discover novel prognostic biomarkers that could guide therapy.

Some studies have indicated that elevated plasma Gd-IgA levels are associated with a worse renal prognosis [5–8], but results are conflicting [9]. Urinary Gd-IgA has been less studied as a biomarker in IgAN patients. Suzuki et al. found that urinary Gd-IgA levels were elevated in patients with IgAN compared to patients with CKD of other causes and that concentrations correlated with the degree of proteinuria [10].

The aim of the present study was to investigate associations between Gd-IgA1 concentrations in serum and urine and disease activity and progression in patients with IgAN. We hypothesized that elevated levels of Gd-IgA1 might be predictive of a worse clinical outcome. For this purpose, we analysed Gd-IgA1 in a well characterized cohort of IgAN patients at our centre with a follow-up time of 5–10 years.

## Materials and methods

### Cohort of patients with IgAN and study design

This is a retrospective single centre cohort study of adult patients (age > 18 years) who underwent a diagnostic kidney biopsy at the Section of Nephrology, Sahlgrenska University Hospital, Gothenburg, Sweden, starting from 2008. Consecutive patients with a clinical indication for kidney biopsy were asked to participate and patients diagnosed with IgAN were included in the present analysis. The study was approved by the regional ethics committee in Gothenburg and all participants, including controls without IgAN (vide infra), gave written informed consent. The study was conducted in accordance with the Helsinki Declaration.

Blood and urine samples were collected at the time of biopsy for routine analyses that included urinary albumin-to-creatinine ratio (UACR) on the first morning void. In addition, serum and urine samples were prepared and stored at -80 °C for later analyses. All patients with a diagnosis of IgAN and sufficient volumes of stored serum and urine from the time of biopsy (n = 40) were identified and included in the present study. Serum and urine from the time of kidney biopsy were analysed for Gd-IgA1 and serum samples were also analysed for total IgA (IgA) and the ratio Gd-IgA1:IgA was calculated. Urinary Gd-IgA1 levels were related to the creatinine concentration and expressed as a ratio (µg/mmol).

Medical records were retrospectively reviewed and clinical data were collected. Data on estimated glomerular filtration rate (eGFR) were collected annually and the average of annual changes in eGFR (ml/min/1.73 m^2^ per year) during follow up was calculated. Estimated GFR was calculated using the CKD-EPI equation [11]. Similarly, average annual changes in UACR (mg/mmol per year) were calculated.

### Analyses of intra-individual changes in Gd-IgA1 during follow up

To assess intra-individual changes in Gd-IgA1 during follow up we contacted all study participants with IgAN in years 2020–2021 and asked for blood and urine samples. Out of 40 study subjects, 19 agreed to submit new samples and in this subgroup the median follow-up time from the diagnostic kidney biopsy was 116 months (range 52 to 161).

### MEST-C scores

MEST-C scores [12] were determined by an experienced pathologist at Sahlgrenska University Hospital. Mesangial proliferation (M1), endocapillary proliferation (E1), and segmental sclerosis (S1) were scored as present or not (M0, E0, S0 respectively) while cortical tubular atrophy/interstitial fibrosis was graded as < 25% (T0), 25–50% (T1) or > 50% (T2). Crescents were graded as absent (C0), < 25% of glomeruli (C1), or > 25% of glomeruli (C2).

### Gd-IgA1 levels in control groups without IgAN

Serum IgA and Gd-IgA1 were analysed in 19 healthy controls. In addition, measurements of serum IgA and serum and urinary Gd-IgA1 were conducted on 21 patients with CKD stage 3 and 4 without IgAN (hypertensive nephropathy [n = 7], diabetic kidney disease [n = 5], polycystic kidney disease [n = 3], chronic tubulointerstitial nephritis [n = 2], and four patient with thin glomerular membrane disease, obstructive nephropathy, membranoproliferative glomerulonephritis, and postinfectious glomerulonephritis, respectively). Controls were matched for gender and were derived from cohorts at our centre that have been characterized previously [13, 14].

### Sample preparation

Urine samples were centrifuged for 10 min at 800 g and 4 °C. The supernatant was collected and stored at -80 °C until analyzed. Blood samples were left at room temperature for 15–30 min and centrifuged at 1,000 g at 4° C for 10 min and serum was collected and stored at -80° C until analysed.

### Gd-IgA1 and total IgA analyses

The analysis of Gd-IgA1 was made with ELISA using the monoclonal KM55 antibody (IBL27600, IBL, Japan). Serum samples were diluted between 1:400 and 1:2000 in supplied EIA buffer. Urine samples were diluted between 1:1 and 1:100. The assay plate was incubated with TMB chromogen substrate and the reaction was stopped after 30 min.

Analysis of IgA was performed using an ELISA kit (ab137980, Abcam, Cambridge, UK). Serum samples were diluted between 1:100000 and 1:20000 in assay buffer according to the manufacturer’s instructions. The assay plate was incubated with TMB chromogen substrate for 30 min.

Readouts for both Gd-IgA1 and IgA were performed on a SpectraMax i3 microplate spectrophotometer (Molecular Devices, San Jose, CA) at 450 nm. Results were calculated automatically using a 4-parameter standard curve equation.

### Biochemical routine analyses

Clinical routine analyses were carried out by standard laboratory methods at the Department of Clinical Chemistry at Sahlgrenska University Hospital (SWEDAC approved according to European norm 45,001). Plasma creatinine was measured with an enzymatic assay using Roche’s cobas system. Urinary creatinine was measured on samples diluted 1:25 with an ELISA based detection kit (Invitrogen, USA) according to the manufacturer’s instructions.

### Statistical analyses

Continuous variables were expressed as either means ± standard deviation or medians with interquartile range (IQR) and compared with Mann-Whitney U or Kruskal-Wallis test as appropriate. Bonferroni correction was used when multiple pairwise comparisons were performed. Categorical variables were expressed as proportions and compared using Chi-square and Fisher’s exact test. Correlations were analysed with Spearman’s rho coefficient. For the analysis of paired data Wilcoxon sign-rank test was used. A p-value < 0.05 was considered statistically significant. Statistical analyses were performed using software SPSS (IBM SPSS Statistics for Windows, Version 28.0.1.1 Armonk, NY, USA).

## Results

### Baseline characteristics at time of kidney biopsy (Table 1)

**Table 1:** Patient characteristics

		IgAN (n = 40)	CKD non-IgAN (n = 21)	Healthy controls (n = 19)	p-value
Age, years		42 (15)	51 (12)	53 (12)	NA
Men (%)		29 (73)	17 (81)	14 (74)	NA
Diabetes (%)		2 (5)	5 (24)	0 (0)	NA
ACEI or ARB (%)		14 (35)	16 (76)	0 (0)	NA
Immunosuppressive treatment (%)		3 (7.5)	0 (0)	0 (0)	NA
Serum creatinine, µmol/L		125 ± 62	199 (90)	81 (6)	NA
eGFR, ml/min/1.73m^2^		68 (28)	36 (16)	81 (7)	NA
SBP, mmHg		136 (20)	132 (18)	119 (12)	NA
DBP, mmHg		84 (13)	82 (9)	72 (8)	NA
UACR, mg/mmol		115 (114)	64 (123)	0.2 (< 0.1)	NA
Serum IgA, µg/mL		2932 (2177–3881)	1867 (1560–2264)	2040 (1513–2473)	p = 0.001
Serum Gd-IgA1, µg/mL		6.0 (3.7-10-3)	2.0 (1.2–4.9)	2.0 (1.3–2.4)	p < 0.001
Serum Gd-IgA1:IgA, x10^− 3^		2.1 (1.3–3.8)	1.2 (0.8–2.5)	0.9 (0.6–1.4)	p < 0.001
Urinary Gd-IgA1 µg:creatinine, µg/mmol		25 (4–53)	7 (3–12)	NM	p = 0.049

Values are presented as mean ± SD, median (IQR) or number of patients (%). P-values are for the Kruskal-Wallis test. IgAN,IgA nephropathy; IgA, Immunoglobulin A; ACEI, angiotensin-converting enzyme inhibitor; ARB, angiotensin receptor blocker; UACR, urinary albumin-to-creatinine ratio, Gd-IgA1, galactose deficient IgA1; eGFR, estimated glomerular filtration rate; SBP, systolic blood pressure; DBP, diastolic blood pressure; NA, not applicable; NM, not measured

**Fig. 1:**
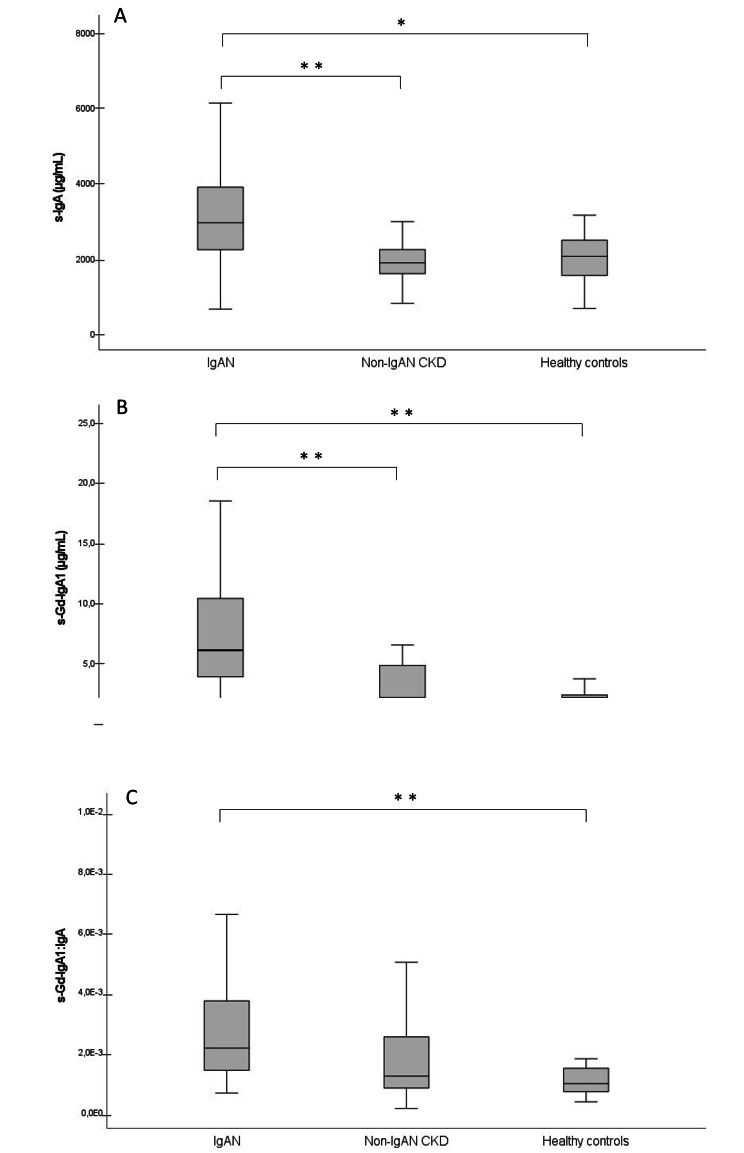
S-IgA (A), Gd-IgA1 (B) and Gd-IgA1:IgA (C) at time of kidney biopsy IgAN (n = 40), Non IgAN CKD patient (n = 21), Healthy controls (n = 19) * denotes p < 0.05 and ** p < 0.001 between groups. Abbreviations: IgA: Immunoglobulin A, IgAN: IgA nephropathy, CKD: chronic kidney disease, Gd-IgA1: galactose deficient IgA1.

Patients with IgAN were younger than controls and had higher eGFR and UACR compared to patients with non-IgAN CKD. Serum IgA and Gd-IgA1 were significantly elevated in patients with IgAN vs. patients with non-IgAN CKD and healthy controls (Fig. 1). Serum Gd-IgA1:IgA differed significantly between groups with highest values in patients with IgAN. Serum Gd-IgA1:IgA was significantly elevated in patients with IgAN vs. healthy controls (p = 0.001) but did not reach statistical significance vs. patients with non-IgAN CKD (p = 0.053) (Fig. 1). These results were not significantly altered after exclusion of the three patients on prednisolone treatment from the analysis.

Serum levels of IgA, Gd-IgA1, and Gd-IgA1:IgA in patients with non-IgAN CKD were comparable to those in healthy controls. The urinary Gd-IgA1:creatinine was significantly increased in patients with IgAN vs. patients with non-IgAN CKD (p = 0.049). At the time of biopsy three patients with IgAN were on immunosuppressive therapy (all with prednisolone). A total of six IgAN patients (15%) were treated with immunosuppressive drugs at some point during the study period.

### Associations between MEST-C scores and UACR, eGFR and Gd-IgA1 at time of kidney biopsy

There were no statistically significant differences in eGFR between patients with M1 vs. M0, E1 vs. E0 or S1 vs. S0 (data not shown). Patients with T1 had lower eGFR compared to patients with T0 (51 [IQR 49–65] vs. 86 [IQR 70–115] mL/min/1.73m^2^, p < 0.001 and patients with T2 had lower eGFR than patients with T1 (34 [23–49] vs. 51 [IQR 49–65] mL/min/1.73m^2^, p = 0.001).

Patients with M1 had elevated UACR compared to those with M0 (227 [IQR 107–297] vs. 54 [IQR 18–144] mg/mmol, p = 0.003) and patients with T2 had elevated UACR vs. those with T0 (226 [IQR 124–338] vs. 33 [IQR 14–83] mg/mmol, p = 0.003).

There were no statistically significant differences in serum Gd-IgA1 concentrations between patients with different MEST-C scores (Table 2). No patient showed C2 lesions. Similarly, there were no statistically significant differences in serum IgA, or Gd-IgA1:IgA, between patients with different MEST-C scores (data not shown).

Urinary Gd-IgA1:creatinine was elevated in patients with T2 scores vs. those with T0 (47.6 [IQR 26-71.4] vs. 6.5 [IQR 8.8–26.1] µg/mmol, p = 0.047). No additional, statistically significant, differences were found in urinary Gd-IgA1:creatinine between patients with different MEST-C scores (data not shown).

**Table 2:** S-Gd-IgA1 in patients with IgAN (n = 40) according to MEST-C subgroups

		s-Gd-IgA1 (µg/mL)	(n)	p-value
M0		6.9 (3.6–11.0)	30	
M1		5.5 (4.2–8.9)	10	0.59
E0		5.6 (3.8–9.9)	33	
E1		8.8 (3.6–12.7)	7	0.31
S0		8.3 (3.2–12.5)	13	
S1		5.6 (3.9–9.5)	27	0.84
T0		4.6 (3.5–9.6)	18	
T1		8.8 (5.4–11.4)	15	
T2		5.6 (4.8–8.3)	7	0.27
C0		6.0 (4.0-10.5)	36	
C1		5.4 (3.2–8.1)	4	0.47

Values are median (IQR). IgA, Immunoglobulin A; IgAN, IgA nephropathy; Gd-IgA, galactose deficient IgA. For definitions of MEST-C scores see “Methods”. P values are for the Mann-Whitney U and Kruskal-Wallis tests.

### Correlation analyses at time of kidney biopsy in patients with IgAN (n = 40)

**Fig. 2:**
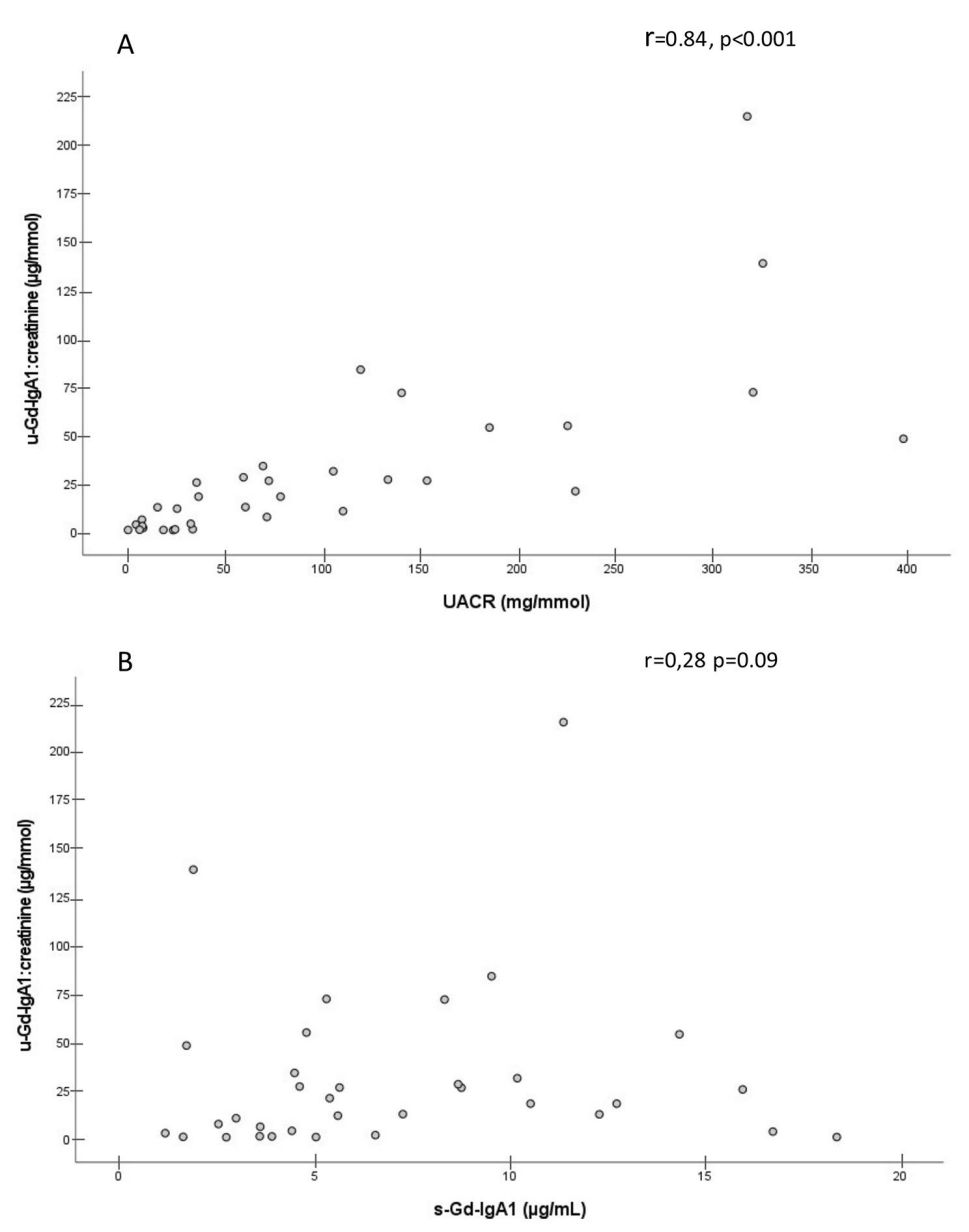
Correlations between u-Gd-IgA1:creatinine UACR (A), and s-Gd-IgA1 (B) at time of kidney biopsy (n = 40). Abbreviations: IgA: Immunoglobulin A, Gd-IgA1: galactose deficient IgA1, UACR: urine albumin:creatinine ratio. r, Spearman’s correlation coefficient

There was a significant inverse correlation between UACR and eGFR (r=-0.39, p = 0.012). Neither serum Ig-A, Gd-IgA1, Gd-IgA1:IgA, IgA/C3, nor Gd-IgA1:C3 showed statistically significant correlations to either eGFR, UACR, systolic or diastolic BP, or age (values not shown). Urinary Gd-IgA1:creatinine showed a statistically significant positive correlation to UACR but not to serum Gd-IgA1 (Fig. 2). Both UACR (r = 0.47, p < 0.001) and urinary Gd-IgA1:creatinine (r = 0.47, p < 0.001) were significantly correlated to systolic blood pressure.

### Prognostic role of serum Gd-IgA1 at time of kidney biopsy in patients with IgAN (n = 40)

The median follow-up time after kidney biopsy was 78 months (IQR: 60–120 months). Median ΔGFR per year was − 2.1 (IQR − 3.8 - -0.6) mL/min/1.73m^2^. Median ΔUACR per year during follow up was − 5 (IQR − 19.3 − 0.25) mg/mmol. There was a statistically significant correlation between UACR at baseline and ΔUACR per year (r=-0.76, p < 0.001) but not with ΔGFR per year (r=-0.29, p = 0.072).

There was no statistically significant correlation between serum Gd-IgA1 and ΔGFR per year during follow up (r=-0.14, p = 0.39). In addition, neither serum IgA (r = 0.21, p = 0.19), Gd-IgA1:IgA (r=-0.04, p = 0.8), nor Gd-IgA1:C3 (r=-0.072, p = 0.74) correlated significantly with ΔGFR during follow up. These results were not significantly altered after exclusion of the three patients on prednisolone treatment from the analysis.

Similarly, there were no statistically significant correlations between serum Gd-IgA1, IgA or Gd-IgA1:IgA and ΔUACR per year during follow up (values not shown). Furthermore, there were no statistically significant correlations between serum Gd-IgA1, IgA or Gd-IgA1:IgA at baseline and changes in UACR or eGFR during the first 6 months after kidney biopsy (values not shown).

### Prognostic role of urinary Gd-IgA1:creatinine at time of kidney biopsy in patients with IgAN (n = 40)

There was no statistically significant correlation between urinary Gd-IgA1:creatinine and ΔGFR per year during follow up (r=-0.22, p = 0.16). Urinary Gd-IgA1:creatinine showed a significant inverse correlation to ΔUACR during follow up (r=-0.67, p < 0.001).

### Intra-individual changes in s erum and urinary Gd-IgA1 during follow-up in patients with IgAN (n = 19)

**Fig. 3:**
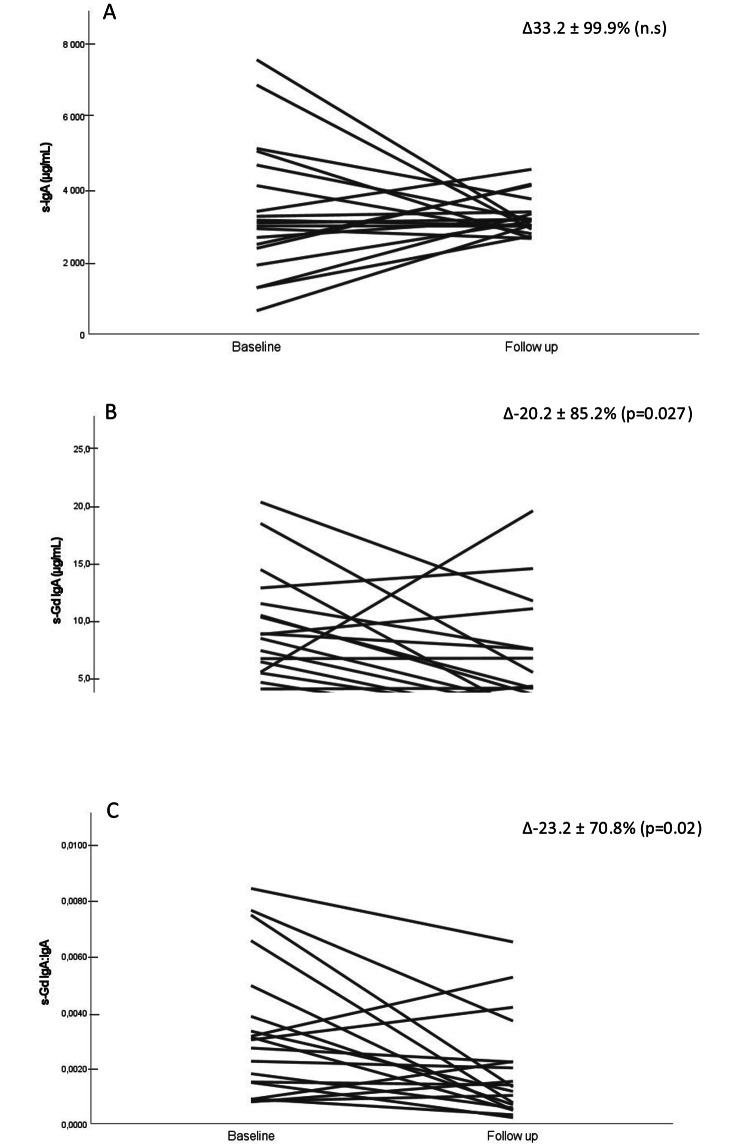
Individual changes in serum IgA (A), GdIgA1 (B), and Gd-IgA1/IgA (C) between baseline and at follow up. Median follow up time was.116 months, (n = 19). Abbreviations: IgA: Immunoglobulin A, Gd-IgA1: galactose deficient IgA1.

**Fig. 4:**
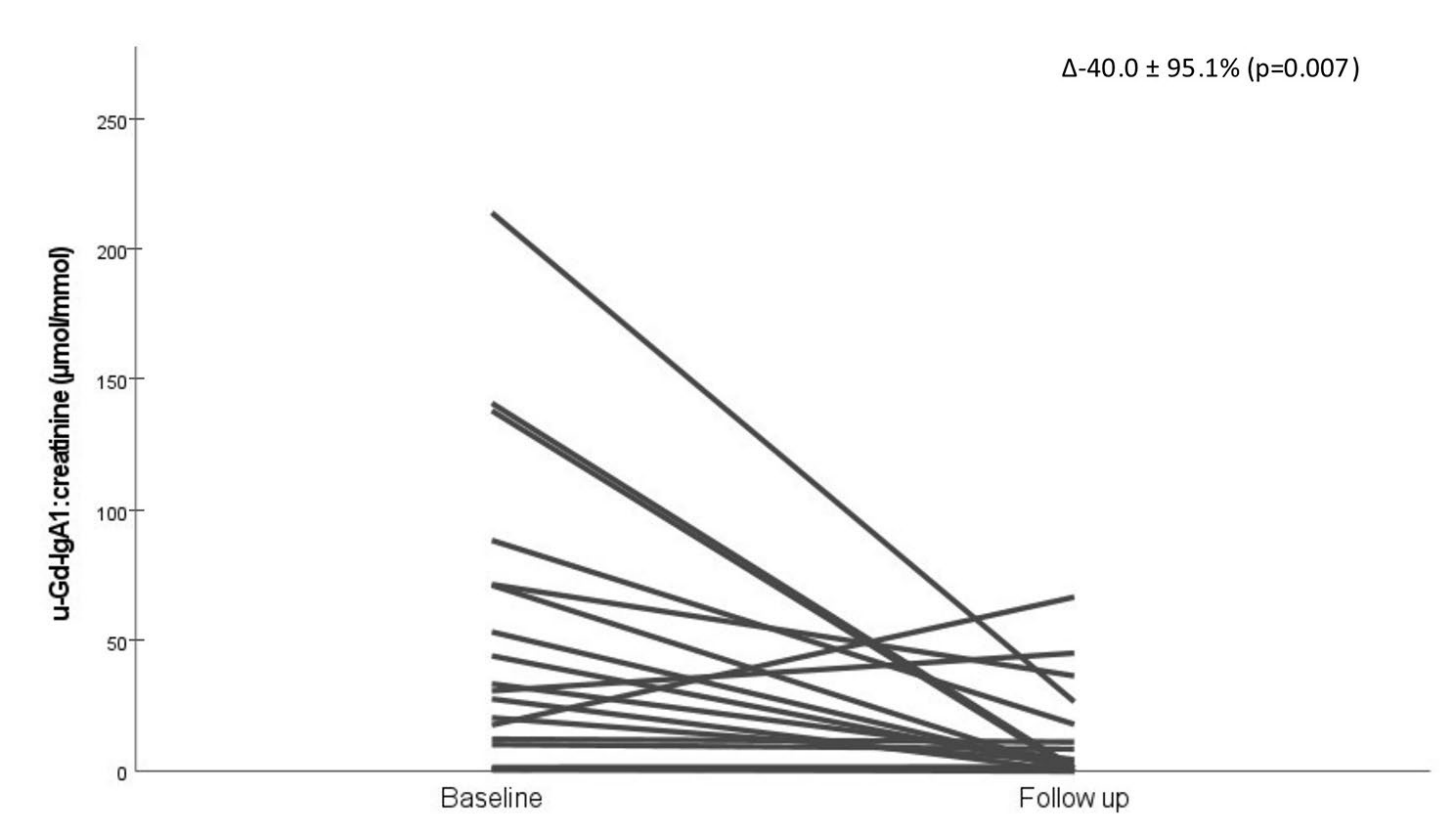
Individual changes in urinary Gd-IgA1:creatinine between baseline and at follow up. Median follow up time 116 mån, (n = 19). Abbreviations: Gd-IgA1: galactose deficient IgA1.

Nineteen patients had blood and urine samples taken after a median follow-up time of 116 months after the diagnostic kidney biopsy. Serum IgA concentrations did not change significantly during this interval (Fig. 3). However, both serum Gd-IgA1 and Gd-IgA1:IgA decreased significantly with time (Fig. 3). Similarly, urinary Gd-IgA1:creatinine was significantly reduced at follow-up (Fig. 4).

We assessed whether changes in serum Gd-IgA1 or Gd-IgA1:IgA were associated with changes in kidney function during the corresponding follow up period. However, changes in serum Gd-IgA1 and Gd-IgA1:IgA did not correlate significantly to ΔGFR or ΔUACR. Changes in urinary Gd-IgA1:creatinine during follow up correlated significantly to ΔUACR (r = 0.60, p = 0.008) but not to ΔeGFR.

Four out of 19 patients had received immunosuppressive therapy during the follow up period. After excluding these four individuals from the analyses reductions in serum Gd-IgA1 and Gd-IgA1:IgA over time were no longer statistically significant (p = 0.125 for both analyses).

## Discussion

The main finding of the present study was that although patients with IgAN had elevated serum concentrations of Gd-IgA1 at the time of kidney biopsy compared to control groups, Gd-IgA1 levels were not associated with disease activity. Furthermore, serum Gd-IgA1 levels did not show significant correlations to changes in eGFR or UACR during a median follow up time of 6.5 years indicating poor prognostic utility.

Previous studies, in which the same monoclonal antibody for Gd-IgA1 detection was used as in the present study, have consistently shown that patients with IgAN have elevated levels of serum Gd-IgA1 compared to patients with non-IgAN CKD and healthy controls [5, 7, 9]. Our results are in line with these data and support a role for Gd-IgA1 in the pathophysiology of IgAN. In addition, serum concentrations of Gd-IgA1 in patients with IgAN in the present study were in good agreement with levels from previous studies [5, 15]. Considering the important role of Gd-IgA1 in the pathophysiology of IgAN [3] we hypothesized that serum Gd-IgA1 concentrations, at the time of diagnostic biopsy, would correlate to disease activity and progression. The results of the present study did not show any such correlations indicating no prognostic value of serum Gd-IgA1 in our cohort of patients. Previous studies that examined correlations between serum Gd-IgA1 levels and disease activity and progression have shown diverse results. Wada et al. [7] measured serum Gd-IgA in 111 patients with IgAN and found that each 5 µg/mL increase in Gd-IgA produced a near 40% higher risk of a 30% reduction in eGFR during a follow up time of approximately 5 years. However, like in the present study, they did not find a significant correlation between serum Gd-IgA1 and eGFR at baseline. Kim et al. [5] studied 230 patients in Korea with IgAN and found a weak inverse correlation between serum Gd-IgA1 and eGFR at baseline. These authors also demonstrated that serum Gd-IgA1 levels predicted CKD progression defined as a 25% reduction in eGFR, or a decline in eGFR category, from baseline. Approximately 40% of patients with serum Gd-IgA1 ≥ 11.31 µg/mL were identified as progressors vs. 20% of patients with lower serum Gd-IgA1 concentrations. On the contrary, Bagchi et al. [9] did not find a significant difference between patients with high and low serum Gd-IgA1 values (cut off value 7.98 µg/mL) in CKD progression defined as a 30% reduction in eGFR, ESRD or death. In addition, and in line with our results, these authors did not find statistically significant correlations between serum Gd-IgA1 concentrations and MEST scores [9].

There are many methodological differences between the present study and the abovementioned studies that may explain discrepant results and make comparisons difficult. First, whereas we assessed annual changes in eGFR and UACR as measures of disease progression, the other studies examined a binary endpoint (progressors or non-progressors). In addition, the duration of the follow up period differed between studies. It is our view that it is unsuitable to have a 25–30% reduction in eGFR as an endpoint for progression especially for observational data. A reduction in eGFR of this magnitude may not reflect disease activity and progression. Secondly, the proportion of patients treated with immunosuppressants was very different in the studies. In the present study only six patients (15%) were treated with immunosuppressants at some point during the study period whereas almost 50% of patients in the study by Kim et al. received immunosuppressive therapy [5]. Thirdly, the clinical indication for performing a diagnostic kidney biopsy when IgAN is suspected varies a lot between countries, regions, and individual nephrologists [16]. Hence, biopsies may have been performed at different phases of the disease course in the abovementioned studies. In addition, the studies vary in size and our study has some limitations in terms of small size.

In the current study, as in most earlier studies [7, 9], there were no significant correlations between serum Gd-IgA1 at time of biopsy and markers of disease activity such as eGFR, UACR or MEST-C scores. A likely explanation for these findings is that the diagnosis of IgAN often is delayed and biopsies are presumably carried out long after disease onset. In support of this, most patients in the present study (22 out of 40 patients) had T1 or T2 scores in their diagnostic biopsy. Hence, it is possible that serum Gd-IgA1 concentrations are related to disease activity earlier in the course of the disease and that other pathophysiological mechanisms such as glomerular complement activation and mediators of inflammation and fibrosis [17, 18] are more important at the time of biopsy.

In the present study measurements of serum Gd-IgA1 were repeated in 19 patients after an average of approximately 10 years. Although serum levels of total IgA were not significantly changed both Gd-IgA1 and the Gd-IgA1:IgA ratio were significantly reduced compared to the time of biopsy. After exclusion of the four patients that had been subjected to immunosuppressive therapy during the study period the reduction in serum Gd-IgA1 was no longer statistically significant. However, in 9 out of 15 patients who were not treated with immunosuppressive agents serum Gd-IgA1 and Gd-IgA1:IgA decreased during follow up. Hence, our results suggest that serum levels of Gd-IgA1 decrease over time in most patients with IgAN and even in those who do not undergo immunosuppressive therapy. This should be considered in situations when serum levels of Gd-IgA1 are used to monitor treatment effects.

We hypothesized that urinary Gd-IgA1:creatinine might reflect disease activity in IgAN at the tissue level and therefore could be a useful disease-specific biomarker. However, urinary Gd-IgA1:creatinine showed a strong positive correlation to UACR at baseline and changes in Gd-IgA1:creatinine during follow up paralleled changes in UACR. In addition, the correlation between UACR and urinary Gd-IgA1:creatinine was much stronger than the correlation between serum Gd-IgA1 and urinary Gd-IgA1:creatinine. These findings suggest that increased urinary Gd-IgA1:creatinine might be a consequence of glomerular injury and a sign of unselective proteinuria in patients with IgAN. The collinearity between urinary Gd-IgA1:creatinine and UACR makes the interpretation of Gd-IgA1:creatinine difficult and questions its use as a biomarker. Our data support previous result of Suzuki et al. [10] who demonstrated that urinary Gd-IgA1 correlated with the level of proteinuria in a cohort of 207 patients with IgAN. Further studies are needed to determine if urinary analyses of Gd-IgA1 brings additive clinical value beyond those of serum measurements.

Strengths of the present study were that our cohort of IgAN patients was well characterized, and that the follow-up time was long. The main limitations were the small number of patients and the retrospective study design. Clearly, prospective, multi-center, studies with much larger number of patients are needed to conclusively determine the prognostic role of Gd-IgA in IgAN. Another limitation of the present study was that patients treated with prednisolone were included in the study. However, the number of patients treated with prednisolone was small and exclusion of these patients from our main analyses had no significant effects on the results.

## Conclusion

Patients with IgAN have elevated serum levels of Gd-IgA1 and Gd-IgA1:IgA compared to patients with non-IgAN CKD and healthy controls. However, no significant correlation between serum Gd-IgA1 and Gd-IgA1:IgA and disease activity or progression during a 6.5 year follow up could be shown. Hence, the clinical utility of Gd-IgA1 as a prognostic biomarker remains to be proven and there is a need for further prospective studies.

## Data Availability

The data underlying this article will be shared on reasonable request to the corresponding author.

## List of Abbreviations

*CKD*: Chronic kidney disease
*eGFR*: Estimated Glomerular Filtration Rate
*ESRD*: End stage renal diseaseI
*Gd-IgA1*: Galactose-deficient IgA1
*IgAN*: IgA nephropathy
*MEST-C score*: Mesangial proliferation, endocapillary proliferation, segmental sclerosis and crescents
*UACR*: Urine albumin:creatinine.
